# Whole-orbit radiomics: machine learning-based multi- and fused- region radiomics signatures for intravenous glucocorticoid response prediction in thyroid eye disease

**DOI:** 10.1186/s12967-023-04792-2

**Published:** 2024-01-13

**Authors:** Haiyang Zhang, Mengda Jiang, Hoi Chi Chan, Huijie Zhang, Jiashuo Xu, Yuting Liu, Ling Zhu, Xiaofeng Tao, Duojin Xia, Lei Zhou, Yinwei Li, Jing Sun, Xuefei Song, Huifang Zhou, Xianqun Fan

**Affiliations:** 1 Department of Ophthalmology, Shanghai Ninth People’s Hospital, Shanghai Jiao Tong University School of Medicine, Shanghai, China; 2 Shanghai Key Laboratory of Orbital Diseases and Ocular Oncology, Shanghai, China; 3 Department of Radiology, Shanghai Ninth People’s Hospital, Shanghai Jiao Tong University School of Medicine, Shanghai, China; 4 School of Health Science and Engineering, University of Shanghai for Science and Technology, Shanghai, China

**Keywords:** Thyroid eye disease, MRI, Radiomics analysis, Intravenous glucocorticoid, Response prediction, Multi-organ segmentation

## Abstract

**Background:**

Radiomics analysis of orbital magnetic resonance imaging (MRI) shows preliminary potential for intravenous glucocorticoid (IVGC) response prediction of thyroid eye disease (TED). The current region of interest segmentation contains only a single organ as extraocular muscles (EOMs). It would be of great value to consider all orbital soft tissues and construct a better prediction model.

**Methods:**

In this retrospective study, we enrolled 127 patients with TED that received 4·5 g IVGC therapy and had complete follow-up examinations. Pre-treatment orbital T2-weighted imaging (T2WI) was acquired for all subjects. Using multi-organ segmentation (MOS) strategy, we contoured the EOMs, lacrimal gland (LG), orbital fat (OF), and optic nerve (ON), respectively. By fused-organ segmentation (FOS), we contoured the aforementioned structures as a cohesive unit. Whole-orbit radiomics (WOR) models consisting of a multi-regional radiomics (MRR) model and a fused-regional radiomics (FRR) model were further constructed using six machine learning (ML) algorithms.

**Results:**

The support vector machine (SVM) classifier had the best performance on the MRR model (AUC = 0·961). The MRR model outperformed the single-regional radiomics (SRR) models (highest AUC = 0·766, XGBoost on EOMs, or LR on OF) and conventional semiquantitative imaging model (highest AUC = 0·760, NaiveBayes). The application of different ML algorithms for the comparison between the MRR model and the FRR model (highest AUC = 0·916, LR) led to different conclusions.

**Conclusions:**

The WOR models achieved a satisfactory result in IVGC response prediction of TED. It would be beneficial to include more orbital structures and implement ML algorithms while constructing radiomics models. The selection of separate or overall segmentation of orbital soft tissues has not yet attained its final optimal result.

**Supplementary Information:**

The online version contains supplementary material available at 10.1186/s12967-023-04792-2.

## Background

Thyroid eye disease (TED), also known as thyroid-associated ophthalmopathy (TAO) or Graves’ orbitopathy (GO), is the most common autoimmune orbital disease that affects 25–40% of patients with Graves’ disease and other thyroid disorders [1, 2].Based on the immune status and disease duration, the pathogenesis of TED can be divided into two phases: an active phase and an inactive phase [1, 2]. With TED progression, lesions develop in all orbital soft tissues [3]. Additionally, TED could be classified into mild, moderate-to-severe, or sight-threatening, based on the evaluation of its clinical manifestations, such as visual acuity, proptosis, and upper eyelid retraction [1]. Intravenous glucocorticoid (IVGC) therapy is the routinely recommended first-line treatment for active and moderate-to-severe TED, offering potent anti-inflammatory effects that could alleviate extraocular muscles (EOMs) edema and orbital lipid hyperplasia [1, 4, 5]. However, the therapy inevitably brings about risks and can result in side effects, such as hypertension, hyperglycemia, and osteoporosis [6–8]. Therefore, proper implementation of IVGC therapy is crucial to achieve maximum benefit and avoid ineffectiveness.

The clinical activity score (CAS) has been used to classify the activity of TED in patients and prescribe IVGC therapy in those with an active status (CAS ≥ 3) [1, 2, 4]. However, CAS does not provide precise prediction, since it is only the record of an ocular inflammatory manifestation and the conceived painfulness, but the pathologic lesions in the posterior orbit are overlooked. In a previous study, based on the application of CAS as a criterion, 38·46% active TED patients (CAS ≥ 3) were determined to be unresponsive to IVGC, whereas 45·45% of the inactive patients (CAS < 3) turned out responsive [9]. Due to its ability to reveal alterations throughout the orbital soft tissues, magnetic resonance imaging (MRI) has been increasingly utilized for TED examination, which effectively contributes markedly to disease activity assessment and therapy response prediction [10, 11]. T2-weighted imaging (T2WI) is a commonly used MRI sequence in clinical applications that provides anatomical and metabolic information of soft tissues [12, 13]. The pathogenesis of the orbital tissues in TED could be clearly revealed on T2WI, characterized by inflammatory edema, chronic fibrosis, and fatty degeneration [12, 13]. Despite the certain predictive value of signal intensity ratio (SIR) or other simple metrics on T2WI for IVGC therapy response, its effectiveness was found to be limited due to insufficient exploitation of images [14]. Therefore, conventional semiquantitative measurements may not ideally meet the requirement of therapy response prediction.

In recent years, radiomics analysis has emerged as a promising solution to this issue by extracting high-throughput quantitative features for further analysis and model construction [15]. It is widely utilized in the field of oncology for the prediction of macrovascular invasion and recurrence [16, 17]. It was first applied in orbital disease by Duron et al. [18] in 2021 to construct an MRI-derived radiomics model in differentiating benign from malignant orbital lesions. Hu et al. [14] have constructed a radiomics model for IVGC response prediction based on the features extracted from EOMs bellies on T2WI, which behaved better than conventional semiquantitative imaging model (AUC = 0·916 vs. 0·745). However, the potential of radiomics for TED therapy response prediction could be further enhanced. Despite EOMs, other vital structures in the orbit, such as lacrimal gland (LG) [19], orbital fat (OF) [20], and optic nerve (ON) [21], also undergo distinct changes in the pathogenesis of TED. The predictive value of these structures has been confirmed in several imaging studies [22–24]. Therefore, our investigation takes a step further in orbital radiomics analysis by integrating all orbital soft tissues to construct a more accurate and robust radiomics prediction model.

Interestingly, similar strategies, namely multi-regional radiomics, have been explored in other human structures and diseases, which performed superior to single-regional radiomics [25, 26]. To the best of our knowledge, no such techniques have been applied for investigations of the ocular orbit. Indeed, fine segmentation of orbital structures on MRI imaging is a challenging task due to its complicacy and considerable time cost. Hence, our study pioneered in this attempt. In order to process high-throughput data from complex segmentation, various machine learning (ML) algorithms were adopted in our study. Ultimately, we established whole-orbit radiomics (WOR) models for the prediction of IVGC response of patients with active, moderate-to-severe TED and attained satisfactory prediction results.

## Methods

### Patients and clinical evaluations

This manuscript adheres to STROBE guidelines. This retrospective study was approved by our Institutional Review Board (SH9H-2021-T246-2), and the requirement for informed consent was waived. Clinical and radiological data of 127 patients with clinically confirmed active and moderate-to-severe TED who had undergone MRI scans before IVGC treatment were collected from the hospital between June 2017 and June 2021. The inclusion criteria were as follows: (1) Patients aged 18–75 years, without complex systemic disease or other orbital disease; (2) High quality of MRI adequate for radiomics analysis; (3) Bilateral manifestation of TED; (4) Disease duration less than 18 months; (5) No previous orbital decompression surgery or radiotherapy, or administration of IVGC ≥ 1·0 g before MRI scans; (6) Patients received IVGC schedule according to standard EUGOGO guidelines (4·5 g, 12 weeks).

The disease activity was evaluated by seven-point CAS, including: (1) Spontaneous retrobulbar pain; (2) pain on attempted up or down gaze; (3) redness of the eyelids; (4) redness of the conjunctiva; (5) swelling of the eyelids; (6) inflammation of the caruncle and/or plica; and (7) conjunctival edema. Patients with CAS < 3 and inactive orbital MRI were categorized as inactive TED, and those with CAS ≥ 3 and active orbital MRI were categorized as active TED. If the indicated activity of CAS and MRI contradicted, an orbital disease specialist with 20 years of experience made a final judgment. The disease severity was assessed according to EUGOGO guidelines. Moderate-to-severe refers to those who met two or more of the following criteria: (1) lid retraction ≥ 2 mm; (2) moderate or severe soft-tissue involvement; (3) exophthalmos ≥ 3 mm above normal for race and gender; (4) inconstant or constant diplopia; without signs of sight-threatening conditions. Ophthalmic assessments for each eye were performed prior to and after the IVGC treatment schedule, including: (1) evaluation of CAS; (2) lid aperture; (3) exophthalmos assessment with a Hertel exophthalmometer; (4) best corrected visual acuity (BCVA); (5) intraocular pressure (IOP); (6) diplopia score. Thyroid-stimulating hormone receptor antibodies (TRAb) was measured before IVGC treatment. Restoration of euthyroidism was recorded if the thyroid-stimulating hormone, free triiodothyronine, and free thyroxine were within the normal range.

Therapy response of IVGC treatment was assessed within three months after the last administration of IVGC. The definition of “responsive” and “unresponsive” was based on the standard proposed by Bartalena et al. [1] The responsive group included those with an improvement of at least two of the following in one eye after treatment: (1) Reduction of lid aperture ≥ 2 mm; (2) Reduction of exophthalmos ≥ 3 mm; (3) Eye motility with an increase of ≥ 8°; (4) Reduction in five-item CAS (not including spontaneous or gaze-evoked pain) of ≥ 1 point; without concomitant deterioration in the other eye. Deterioration was defined by the occurrence of dysthyroid optic neuropathy (DON) or worsening of at least two of the four components mentioned above. The unresponsive group was composed of those who did not meet the aforementioned criteria.

All patients included were allocated to a training cohort and a test cohort with a proportion of 8:2 using a stratified random splitting method. The flowchart of patient enrollment and the scheme for analysis is presented in Additional file 1 Fig. S1.

### Orbital MRI acquisition

**Fig. 1:**
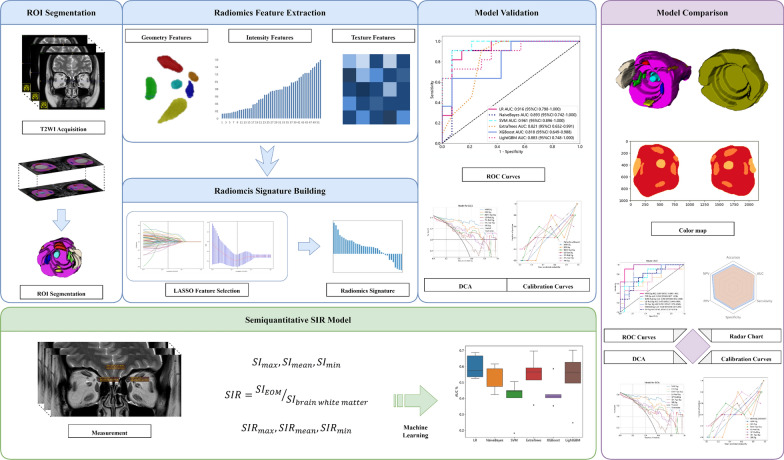
Radiomics workflow

Before the IVGC treatment schedule began, patients were examined using a 3·0 T MRI system (Ingenia CX, Philips Medical Systems) with a 32-channel head coil. During the scan, the patients were placed in the supine position with their eyes closed. Coronal T2-weighted Turbo Spin-Echo with 90° Flip-Back Pulse (T2-DRIVE) imaging was acquired, with the following parameters: repetition time/echo time, 3000/90 ms; field of view, 133·3 133·3 mm^2^; slice thickness, 3·5 mm; slices, 20; gap, 3·85 mm; acquisition matrix, 320 224. Figure 1 depicts the workflow of the radiomics procedure.

### Radiomics analysis

#### ROI segmentation

**Fig. 2:**
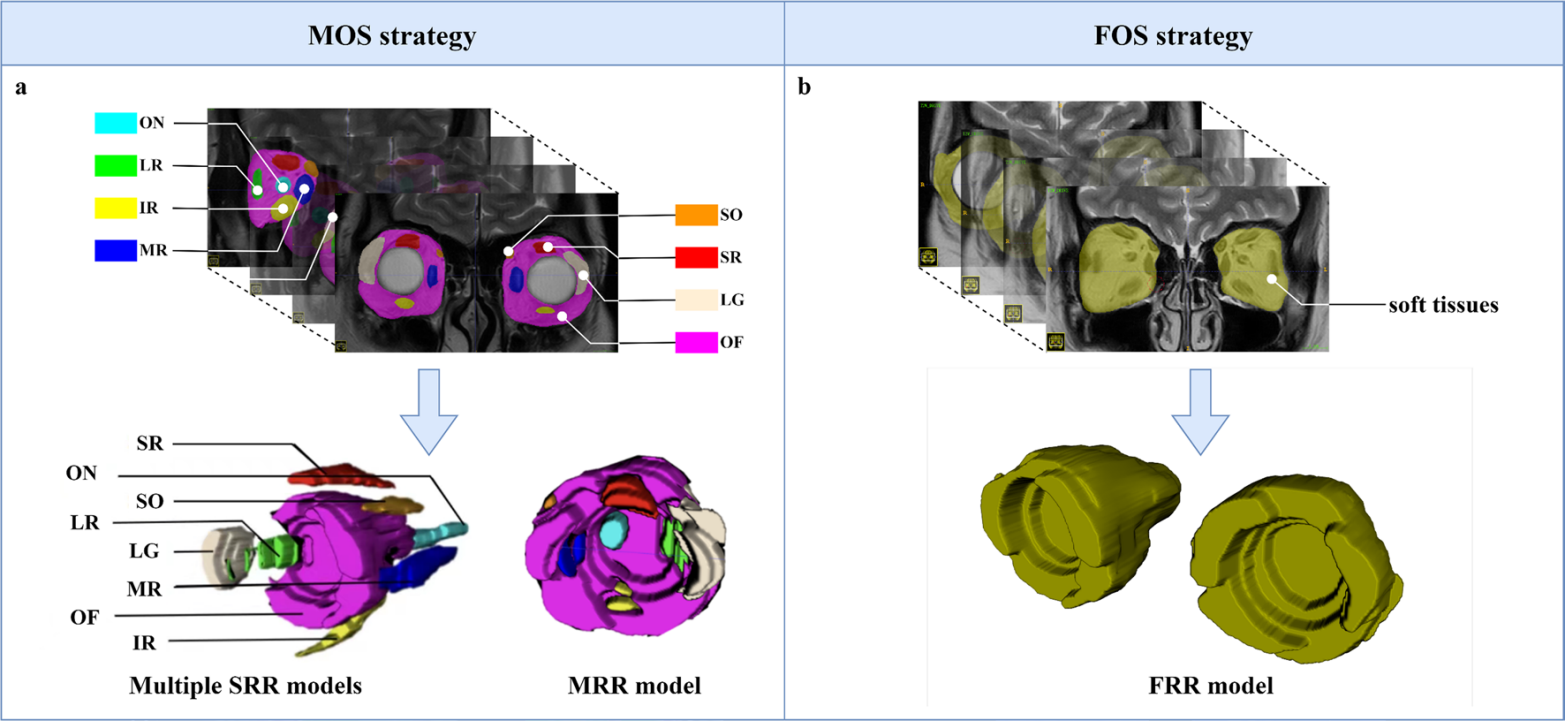
Illustration of the two segmentation strategies on the T2WI. The MOS strategy for the construction of multiple SRR and MRR models is presented in plot **a**, and the FOS strategy for the construction of FRR model is depicted in plot **b**

Regions of interest (ROIs) were manually segmented on coronal T2WI using the ITK-SNAP software (v. 3.6.0; www.itksnap.org). Two methods of ROI segmentation were employed (Fig. 2). The first approach, multi-organ segmentation (MOS) was applied to eight orbital structures, including LG, OF, ON, and separate EOMs: superior rectus (SR), inferior rectus (IR), medial rectus (MR), lateral rectus (LR), and superior oblique (SO). These ROIs were individually contoured using different labels. The contours of each ROIs were drawn slice-by-slice from the emergence of OF in the anterior orbit to the vanish of EOMs in the posterior orbit. Subsequently, four single-regional radiomics (SRR) models were constructed based on different structures (EOMs, LG, OF, and ON), and the dataset comprising all eight labels was later used to develop the multi-regional radiomics (MRR) model. The second approach, namely fused-organ segmentation (FOS) strategy using one single label was also implemented, which regarded all structures including EOMs, LG, OF, and ON as a cohesive unit. A fused-regional radiomics (FRR) model was later built on this basis. For all manual segmentation work, an experienced orbital radiologist (reader 1) viewed each MRI and conducted ROIs segmentations without knowing the disease status of the participants. Each segmented contour was further reviewed by an orbital radiology expert for accuracy. Discussions were held for any disagreement until a consensus on the final decision was reached.

#### Feature extraction

Radiomics features were extracted from ROIs using an in-house feature analysis program implemented in Pyradiomics (http://pyradiomics.readthedocs.io) for all radiomics models (SRR, MRR, and FRR models). Orbital structures from bilateral orbits of the same patient were considered as a unit, and the features were extracted in the meantime. All features were categorized into three groups: (1) geometry features, which described the three-dimensional shape characteristics of the ROIs; (2) intensity features, which described the first-order statistical distribution of the voxel intensities within the ROIs; and (3) texture features, which described the patterns or the second- and higher-order spatial distributions of the intensities. Specifically, to extract texture features, various methods were employed, including the gray-level co-occurrence matrix (GLCM), gray-level run length matrix (GLRLM), gray-level size zone matrix (GLSZM), and neighborhood gray-tone difference matrix (NGTDM) methods.

#### Feature selection

**Fig. 3:**
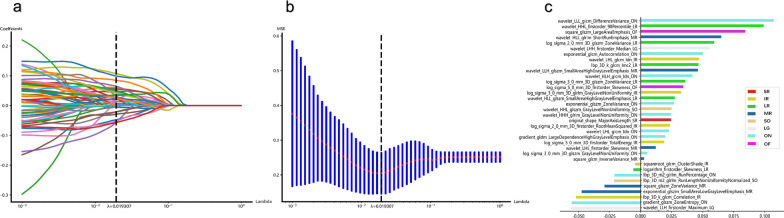
Feature screening for MRR model. Plot shows the coefficients **a** and MSE **b** of LASSO regression model and features selected for model construction **c**

After feature extraction, reproducibility analysis, Mann–Whitney U-test, Spearman's rank correlation, max-relevance, min-redundancy (mRMR), and least absolute shrinkage and selection operator (LASSO) regression were consecutively performed to reduce the feature dimension for the different radiomics models. Initially, 40 cases were randomly chosen (20 of responsive and 20 of unresponsive), and their orbital MRI were segmented by reader 2 in the same manner as reader 1. Inter-reader variation of radiomics features was evaluated by calculating intraclass correlation coefficients (ICC) between the results from reader 1 and reader 2. Only features with ICC > 0·75 were subjected to further analysis. Afterwards, a Mann–Whitney U-test was then employed to identify significant features between responsive and unresponsive groups, only those with a p-value < 0·05 were kept. Then, the Spearman’s rank correlation coefficient was used to identify highly correlated features (Spearman’s correlation coefficient > 0·9), with one of them randomly retained to avoid redundancy. To depict features to the greatest extent, greedy recursive deletion was applied for feature filtering, where the feature with the most redundancy in the current set was deleted each time. Subsequently, to avoid over-fitting and maximizing the correlation between features and target variables, the mRMR algorithm was implemented to select the top eight features for each label. Eventually, the LASSO regression model with tenfold cross test supported by Onekey AI platform was used for signature construction (Fig. 3a, b). The retained features with nonzero coefficients were used for regression model fitting and combined into a radiomics signature (Fig. 3c). The detailed rad score formulae of the models are provided in Additional file 1: Table S1.

#### Radiomics signature construction

SRR, MRR, and FRR models were individually constructed based on the datasets derived from corresponding ROIs as stated above. For all radiomics models, the final selected features were inputted into six robust classification algorithms supported by Onekey AI platform, including logistic regression (LR), NaiveBayes, support vector machines (SVM), extremely randomized trees (ExtraTrees), extreme gradient boosting (XGBoost), and light gradient boosting machine (LightGBM). A five-fold cross-validation was implemented to obtain the final radiomics signatures.

### Semiquantitative measurements and model construction

Semiquantitative measurements on T2WI involved all eight orbital structures, including EOMs, LG, OF, and ON. Two radiologists (reader 1 and reader 2) independently implemented measurements without knowing the disease status of study participants. The signal intensity (SI) of EOMs, LG, and OF was measured by placing polygonal ROIs separately on EOM bellies, LG, and OF, locating the maximum cross-section on the coronal T2WI. The corresponding SI on the anterior and posterior layers of the selected layer of each region of these seven structures were also measured. For measurement of the SI of ON, ROIs were manually segmented on three consecutive layers behind the eyeball, and the surrounding cerebrospinal fluid signal was carefully avoided. For each orbital structure, the maximum, mean, and minimum of SI over the ROI were all extracted, and the final SI_max_, SI_mean_, SI_min_ were recorded as the mean value of SI derived from three consecutive layers. Later, they were normalized to SIR_max_, SIR_mean_, and SIR_min_ using the formula SIR = SI_EOM_/SI_brain white matter_.

Inter-observer variation of measurements between the two observers was assessed by ICC. Then univariate analysis was adopted to test the difference of SIR_max_, SIR_mean_, and SIR_min_ between the responsive and the unresponsive groups. After screening features with P < 0·05, identical six ML algorithms were employed to construct semiquantitative imaging models (SIR models) through five-fold cross-validation.

### Assessment and comparison of different prediction models

The diagnostic performances of the radiomics and semiquantitative imaging models based on different ML algorithms were assessed using their receiver operating characteristic (ROC) curves. For each model, metrics including area under curve (AUC), accuracy, sensitivity, specificity, positive predictive value (PPV), and negative predictive value (NPV) were calculated. Internal validation of the prediction models was performed using an independent set. To compare the largest prediction capacity of different models, the ML algorithm with the highest AUC was finally selected for each model subset for further assessment and comparisons. DeLong’s test was applied to test the difference of diagnostic performance among different models. The calibration curves were depicted to assess the calibration of the prediction models. Decision curve analysis (DCA) was performed to evaluate the clinical usefulness of different models by calculating the net benefits at different threshold probabilities.

### Statistical analyses

All statistical analyses were conducted using Python programming language (version 3.7.6) with the use of SciPy library (1.4.1) and Statsmodels module (v0.11.1). Statistical significance was set at a two-tailed P-value < 0·05. For categorical data, the chi-squared test or Fisher’s exact test was applied to compare the difference between two groups. For numeric data, independent-sample t-test or Mann–Whitney U-test was implemented. Other statistical tools employed for analysis are specified above.

## Results

### Clinical characteristics

Of the 127 enrolled patients, 56 were identified as responsive to IVGC treatment, whereas 71 patients were unresponsive. The clinical characteristics of both groups are presented in Table 1, showing no significant differences in sex, age, or duration time. Univariate analysis revealed significant differences in smoking (P-value = 0·016), diplopia score (P-value = 0·031), CAS (P-value = 0·002), and lid aperture (P-value = 0·031) between the two groups.

**Table 1:** Demographic and clinical characteristics of TED patients and controls

Characteristics	Responsive	Unresponsive	P-value
Sex			0.093
Male	28 (22.4%)	25 (20.0%)	
Female	28 (22.4%)	46 (36.8%)	
Age (year)	47.10 ± 10.32	44.89 ± 11.24	0.891
Disease duration (month)	6.00 (3.00, 12.00)	6.00 (3.50, 14.50)	0.140
Smoking			0.016*
Yes	21 (16.8%)	13 (10.4%)	
No	33 (26.4%)	55 (44.0%)	
Restoration of euthyroidism			0.542
Yes	30 (24.0%)	41 (32.8%)	
No	23 (18.4%)	25 (20.0%)	
TRAb (IU/L)	10.41 ± 12.73	10.82 ± 11.16	0.460
CAS	2.50 (1.50, 3.00)	1.00 (1.00, 3.00)	0.002*
Diplopia score	1.59 ± 1.14	1.30 ± 1.12	0.034*
Exophthalmos (mm)	19.10 ± 2.53	18.24 ± 2.80	0.066
Lid aperture (mm)	10.04 ± 1.46	9.36 ± 1.61	0.031*
IOP (mmHg)	18.41 ± 3.55	18.25 ± 3.18	0.612
BCVA	0.86 ± 0.24	0.87 ± 0.23	0.849

Continuous variables are presented as the mean (± standard deviation) or as the median (interquartile range). Categorical variables are presented as the number (%) and counts

*P-value < 0.05

### Radiomics model construction

#### Single-regional radiomics (SRR) models

**Fig. 4:**
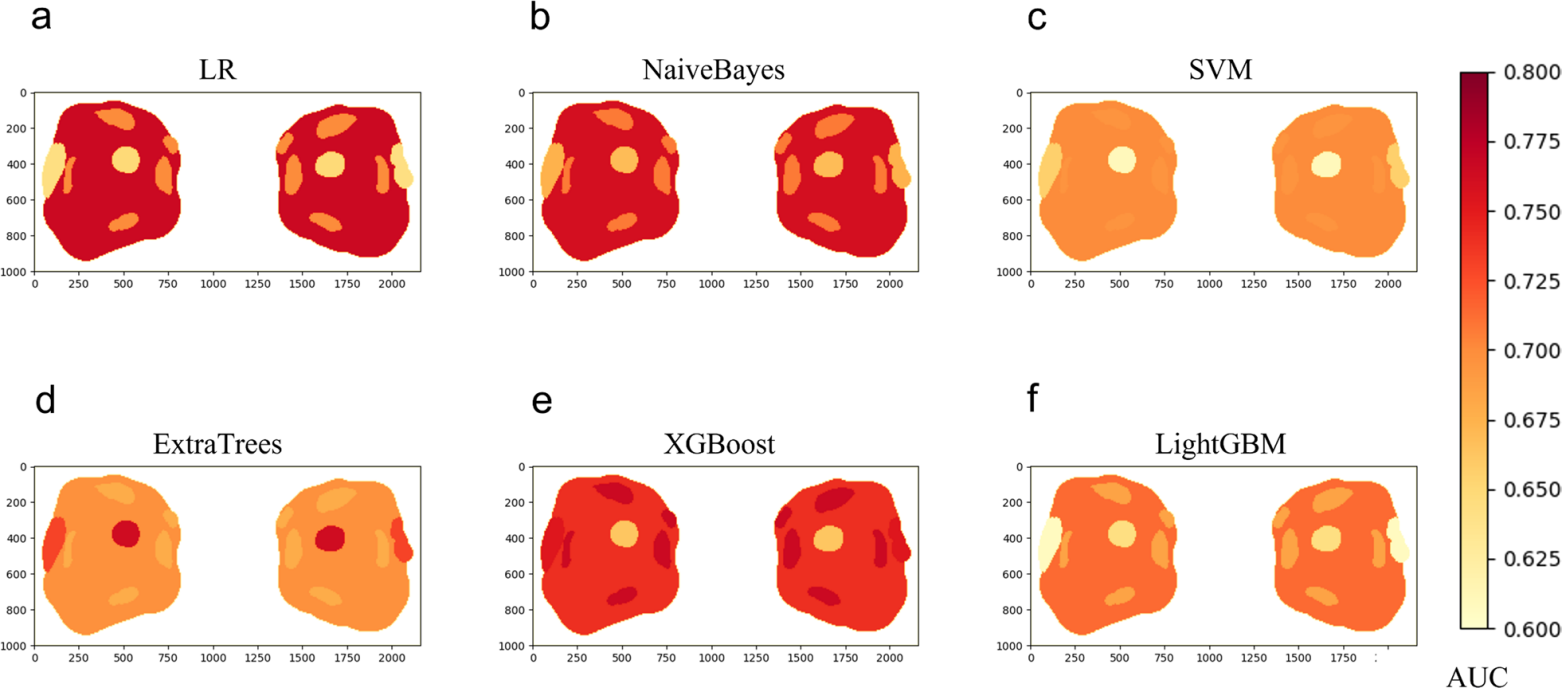
Color maps demonstrating the diagnostic performance of different SRR models (EOM, OF, LG, or ON radiomic models) when utilizing different ML algorithms **a–f**. Colors depicted on each structure represent the AUC of corresponding SRR model based on a specific ML algorithm

Through MOS strategy, 1906 features were respectively extracted from the ROIs of EOMs, LG, OF, and ON. After feature selection, five, eight, five, and seven were finally retained, respectively. For each structure, the corresponding SRR models based on different ML algorithms performed diversely (Fig. 4). For each ML algorithm, EOM radiomics model and OF radiomics model had the best performance. The highest AUC of individual SRR models were achieved by XGBoost on the EOM radiomics model (AUC = 0·766), NaiveBayes on the LG radiomics model (AUC = 0·727), LR on the OF radiomics model (AUC = 0·766), and NaiveBayes on the ON radiomics model (AUC = 0·669), respectively. Details of diagnostic performance of SRR models can be found in Additional file 1: Table S2.

#### Multi-regional radiomics (MRR) models

**Fig. 5:**
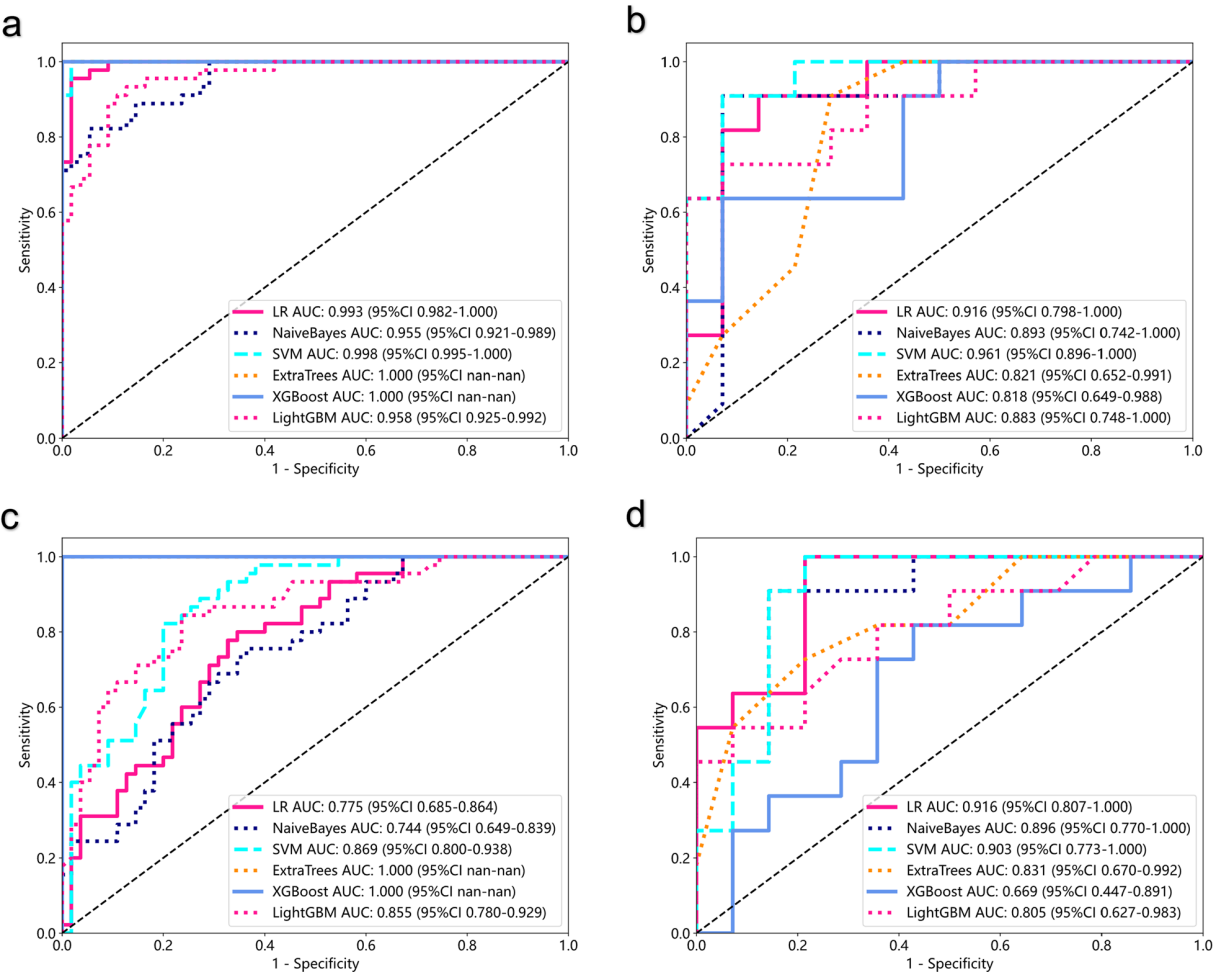
Predictive performance of the MRR (Multi-regional radiomics) and FRR (Fused-regional radiomics) models in the training and test cohorts. The ROC curves of MRR model in training cohort **a** and test cohort **b**; the ROC curves of FRR model in training cohort **c** and test cohort **d**

For the construction of MRR models based on MOS strategy, 15,248 features were extracted from eight independent structures, and 35 were finally retained. Notably, the SVM model achieved remarkable performance, with the highest AUC value of 0·961 in the test cohort. The other models achieved good to excellent AUC values, with LR achieving 0·916, NaiveBayes achieving 0·893, and LightGBM achieving 0·883 (Fig. 5a, b).

#### Fused-regional radiomics (FRR) models

Through FOS strategy, 1906 features were extracted from the cohesive unit of orbital soft tissues and eight were included in the FRR models. All models achieved moderate to good AUC values, with LR achieving 0·916, NaiveBayes achieving 0·896, SVM achieving 0·903 (Fig. 5c, d).

### Semiquantitative model construction

The inter-reader variation of semiquantitative SIRs was found to be good to excellent, with ICCs ranging from 0·766 to 0·893. Results of semiquantitative measurement were shown in Table 2. Models yielded moderate to good results, with most AUC values ranging from below 0·7 to a maximum of 0·760 achieved by the NaiveBayes algorithm (Additional file 1: Fig. S2).

**Table 2:** Semiquantitative SIR values of different orbital soft tissues in TED patients

Characteristics	Responsive	Unresponsive	P-value
LG-SIR_mean_	1.66 ± 0.27	1.61 ± 0.3	0.30
LG-SIR_max_	2.03 ± 0.39	1.93 ± 0.44	0.22
LG-SIR_min_	1.34 ± 0.25	1.31 ± 0.26	0.57
LR-SIR_mean_	1.21 ± 0.29	1.19 ± 0.23	0.43
LR-SIR_max_	1.45 ± 0.30	1.43 ± 0.29	0.59
LR-SIR_min_	0.97 ± 0.22	0.95 ± 0.19	0.47
SR-SIR_mean_	1.37 ± 0.41	1.39 ± 0.37	0.91
SR-SIR_max_	1.70 ± 0.44	1.70 ± 0.44	0.82
SR-SIR_min_	1.05 ± 0.40	1.06 ± 0.35	0.93
MR-SIR_mean_	1.42 ± 0.40	1.28 ± 0.28	0.01*
MR-SIR_max_	1.66 ± 0.44	1.52 ± 0.31	0.02*
MR-SIR_min_	1.20 ± 0.39	1.06 ± 0.29	0.01*
IR-SIR_mean_	1.51 ± 0.39	1.39 ± 0.33	0.03*
IR-SIR_max_	1.80 ± 0.45	1.66 ± 0.39	0.05
IR-SIR_min_	1.22 ± 0.37	1.12 ± 0.29	0.04*
SO-SIR_mean_	1.35 ± 0.28	1.36 ± 0.31	0.74
SO-SIR_max_	1.81 ± 1.29	1.72 ± 0.39	0.42
SO-SIR_min_	0.97 ± 0.26	0.99 ± 0.29	0.06
ON-SIR_mean_	1.25 ± 0.21	1.20 ± 0.21	0.13
ON-SIR_max_	1.69 ± 1.08	1.59 ± 0.8	0.86
ON-SIR_min_	0.96 ± 0.20	0.91 ± 0.18	0.08
OF-SIR_mean_	2.61 ± 0.35	2.60 ± 0.43	0.92
OF-SIR_max_	2.95 ± 0.54	2.96 ± 0.50	0.48
OF-SIR_min_	2.27 ± 0.36	2.27 ± 0.45	0.95

Continuous variables are presented as the mean (± standard deviation)

*P-value < 0.05

### Comparison of different prediction models

**Fig. 6:**
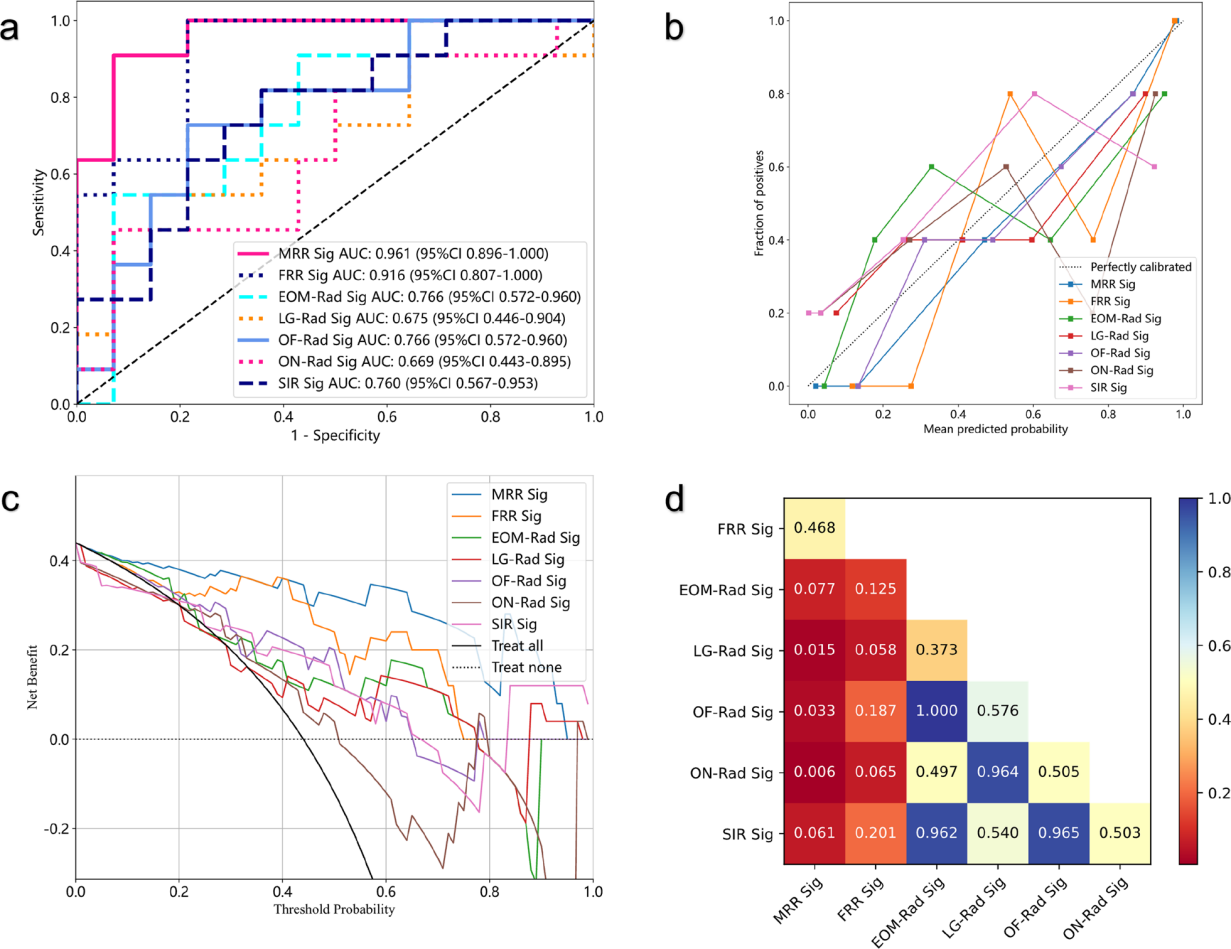
The result and evaluation of prediction models in the test cohort. **a** The ROC curves of different radiomics models and SIR model based on the machine learning algorithms that achieved the highest AUC value. **b** DeLong’s test comparing the diagnostic performance (AUC) of different models. Calibration curves **c** and DCA **d** of different models. *MRR* Multi-regional radiomics, *FRR* Fused-regional radiomics

**Fig. 7:**
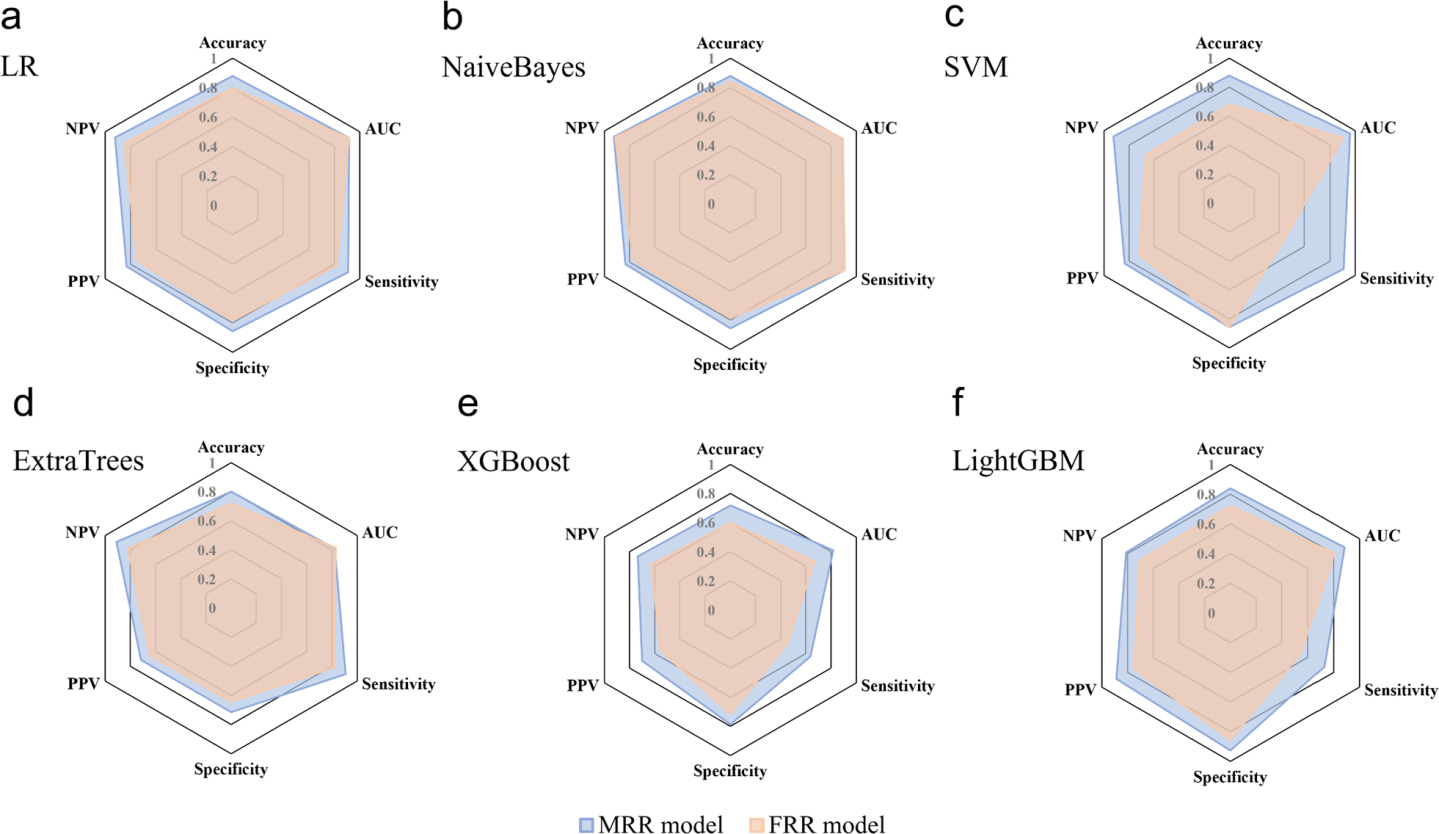
Radar chart of the performance of MRR (Multi-regional radiomics) models and FRR (Fused-regional radiomics) models by using different machine learning algorithms (**a**–**f**)

As is shown in Fig. 6a, radiomics models significantly outperformed semiquantitative imaging model. The WOR models in this study, including MRR (highest AUC = 0·961, SVM) and FRR models (highest AUC = 0·916, LR), had superior performance over all the SRR models, including the formally reported EOM radiomics model (AUC = 0.766) (Fig. 6a). The calibration curves and DCA provided additional supporting evidence to such conclusion (Fig. 6b, c). The MRR model based on SVM had the best performance as regards AUC, calibration, and net benefit. However, further analysis using Delong's test showed that the best performing MRR model based on SVM, and the best performing FRR model based on LR, did not have a significant difference in diagnostic performance (Fig. 6d). Considering the influence of ML algorithms, the comparison of multiple parameters of MRR models and FRR models utilizing the same ML algorithm is shown in Fig. 7. In most cases, the area of the radar chart of MRR is slightly larger than FRR. However, when utilizing NaiveBayes or ExtraTrees, the AUC of FRR is larger than that of MRR.

## Discussion

The preliminary application of radiomics analysis in orbital MRI offers a promising solution to the prediction of IVGC therapy response in TED. Nevertheless, radiomics is still underdeveloped in orbital diseases like TED with deficiency in methodology and practice. In this work, we established the WOR models as a credible and efficient tool to predict IVGC therapy response. The MOS strategy was applied to orbital MRI processing, which included all structures potentially affected in TED. An MRR model (AUC = 0·961) was constructed based on this strategy, reaching a predictive value much superior to SRR models (highest AUC = 0·766) and a conventional semiquantitative imaging model (AUC = 0·760). Besides, we proposed a FOS strategy and constructed an FRR model, as a feasible alternative mode of the WOR models and also achieved a satisfactory result (highest AUC = 0·916). To process high-throughput data, a series of ML algorithms were employed to construct different prediction models and the best was finally chosen. It is highly probable that WOR models will substantially benefit clinical decision-making of TED patients, and that MOS and FOS strategies might bring a new prospect for radiomics research for orbital disease and other disease models.

The MOS strategy has emerged as a highly effective approach in radiomics analysis, as evidenced by a large number of previous studies. For instance, a recent investigation utilized the similar strategy to construct an MRR model that accurately assessed muscle invasion in bladder cancer, with an impressive AUC of 0·931 [25]. Similarly, in cervical cancer, Shi et al. [26] partitioned tumors into two intratumoral subregions to create an MRR model, which were confirmed to be superior to the model based on the whole tumor (AUC = 0·817 vs. 0·562). However, the MOS segmentation is challenging, particularly in the orbital region due to the anatomical complexity. In our study, we incorporated the whole orbital soft tissues associated with the pathogenesis of TED on T2WI. By employing MOS strategy, the MRR model outperformed SRR models that solely relied on a single orbital structure. It serves as another promising application of MOS strategy in radiomics analysis, and the first attempt in orbital setting.

Of the different SRR models, the EOM radiomics model and the OF radiomics model showed relatively good predictive performance, with the highest AUC values of 0·766 for both. As previous studies have suggested, the mechanism of TED pathogenesis is complicated since it affects multiple orbital structures [2]. The pathogenesis of TED is primarily characterized by enlarged and edematous EOMs, making them the major affected structure. Multiple studies revealed that patients who respond well to IVGC have more homogeneous edema within their EOMs, while unresponsive patients exhibit greater tissue complexity and more fibrotic compounds [14, 24, 27, 28]. Similarly, a previous TED radiomics study also constructed the model based on EOMs to predict IVGC therapy response [14]. It is also worth noting that significant differences existed in SIR value of MR and IR between responsive and unresponsive groups in our study. These results prove again that MR and IR are the two primary rectus muscles altered during TED pathogenesis. Nevertheless, they fail to alter the fact that SIR models performed poorly in response prediction compared to MRR and SRR models. Despite EOMs, OF is also a vital morbid structure in the orbits of TED. The majority of patients have enlargement of EOMs or OF, with predominance of one or the other in some [2]. The expansion of OF volume is caused by the accumulation of glycosaminoglycans and adipocytes, which is also the main therapeutic target of IVGC [29, 30]. Previous MRI studies of TED had focused relatively less attention on OF, whereas our earlier studies added evidence to its predictive value in IVGC therapy response [22]. Interestingly, SIR of OF showed no significant difference between the responsive and unresponsive groups, but it is under the premise that the SIR value concentrated on the value determined from a specific point on the structure. However, radiomics model took into account a wider spectrum of features, encompassing geometry, intensity, and texture features. With deeper investigation, detailed information of OF can be extracted and exploited for IVGC response prediction of TED, which was proved to be powerful.

Apart from EOMs and OF, other structures including LG and ON were also of certain predictive value. The highest AUC value of the ON radiomics model was 0·727, while that of the LG radiomics model was 0·675, which was inferior to EOMs and OF. In TED, LG is also affected by immunological disorders in the orbit, characterized by multifocal infiltration of lymphocytes and hyperplasia of adipose tissue [31]. These typical alterations of LG in TED are manifested on T2WI as increased volume and hyperintensity [32]. The herniation of LG has been established to be associated with therapy response of IVGC, demonstrating its contribution to the predictive models [24]. ON is mainly related to visual acuity, concerning the emergence of DON. Interestingly, a retrospective study detected an increased ON T2 value in TED compared with healthy controls [33]. Other studies also indicated a potential correlation between ON and the severity and prognosis of TED. In this investigation, ON was also evidenced to be of predictive value of the IVGC response. Currently, the majority of the studies on activity assessment and response prediction have been focused on EOMs solely, neglecting other affected orbital soft tissues. This has probably attributed to the cognitive deficit, measurability limitations, and time cost. Although the orbital pathologies of different structures are not fully elucidated, and their correlation with radiomics features are scarcely uncovered, we revealed that involving multiple morbid structures in the orbit greatly enhanced the performance of our radiomics models.

By including multiple structures, the MRR model achieved excellent predictive results, but its considerable segmentation efforts may limit the universal application due to the significant time cost. Compared with the EOM radiomics model (total average time, 15·8 min), the performance of the MRR model took much longer (total average time, 25·4 min) for each MRI sample. With the relatively low time cost (total average time, 10·2 min), the semiquantitative imaging model had a moderate predictive value, which was better than those of the ON and LG radiomics models (AUC = 0·760 vs. 0·727 and 0·675, respectively). This outcome was presumably attributable to the incorporation of the whole orbital soft tissue offering more conducive information compared with the single structures. However, the AUC value of semiquantitative imaging model was much inferior to MRR model, which cannot satisfy the requirement of accurate prediction. Therefore, we put forward an alternative WOR model, namely FRR model, which was based on the FOS strategy. When utilizing the same ML algorithms, the performance of FRR model and MRR model was approximate and MRR seemed slightly superior, with the highest AUC value of 0·916 and 0·961 (P-value = 0·468 on DeLong’s test) (Figs. 4a–f, 6d). It is reasonable that MRR outperformed FRR, in that fine segmentation according to priori knowledge is beneficial to image analysis. A recent radiomics investigation revealed that without segmentation masks, feature descriptors encompassed the entire image, which limited their effectiveness in focusing on ROI and leveraging the available prognostic information [34]. This limitation, compounded by noise and the loss of local information regarding size, shape, and location, may have contributed to the slightly lower performance observed in the FRR models. Nevertheless, due to the limited sample size applied in this research, further validation with larger samples is necessary to determine whether the MRR model outperforms the FRR model. However, it is worth noting that the segmentation time cost of the FRR model (total average time of 9·6 min) was only 37·8% of that of the MRR model. This shows the potential of applying the FRR model for IVGC response prediction with higher efficiency. Future explorations of the automatic segmentation of different orbital structures might be of great value to resolving this issue.

In the construction of the prediction models, the ML algorithms played a crucial role for achieving high accuracy and efficiency. However, it is important to consider the suitability of ML algorithms for the input dataset. Our research revealed a shift in the best performing algorithm types from SRR to MRR models. Simpler algorithms such as LR and NaiveBayes worked better in cases of straightforward mapping relationships in ON (Highest AUC = 0·669, NaiveBayes), OF (Highest AUC = 0·766, LR), and LG (Highest AUC = 0·675, NaiveBayes). On the other hand, the XGBoost algorithm showed the highest performance in the EOM dataset (AUC = 0·766) due to its ability to prevent overfitting through shrinkage and generalization features in datasets with multiple labels [35]. Notably, the SVM algorithm attained remarkable results with the highest AUC value of 0·961 in the MRR model. This was due to the fact that SVM was able to recognize and fit valuable underlying mapping effectively when more information was included in the feature datasets [36]. However, the high learning capacity of SVM also made it susceptible to overfitting, leading to poor performance in the semiquantitative imaging model and moderate performance in several SRR models. A deeper investigation of the application of ML algorithms in orbital MRI would provide more solid evidence by using larger datasets, which shall be explored in the future.

Compared with other reported prediction models for IVGC response in TED, the accuracy of our models still needs to be improved. In addition to the potential drawbacks of radiomics analysis, this issue might be attributed to the disunity of the standards for patient enrollment and therapy response evaluations among different studies. The management of TED involves multidisciplinary effort, while many aspects of the diagnosis and treatment are unclear and controversial. For example, the patients in our cohorts met the comprehensive criteria of activity assessment considering CAS and orbital MRI. That is to say, patients with CAS lower than 3 but with actively altered orbital MRI were advised to receive IVGC therapy in our center but were excluded in other centers. This significantly affected the treatment outcome. In addition, the determination of “responsive” or “unresponsive” to anti-inflammatory treatment in TED varied markedly from one study to another. In the present investigation, we adopted a well-recognized evaluation standard proposed by Bartalena et al. [1], integrating four important items of clinical presentations in a composite index. In former studies, usually an eye is perceived as a research object, while in our study, a patient with bilateral eyes were perceived as a research object. This makes our results more feasible for clinical practice. TED clinical management and research work urgently need standardization of evaluation, diagnosis, and treatment.

The present study is a novel attempt to implement the concept of MOS/FOS and MRR/FRR in orbital MRI processing. However, it is only a preliminary exploration and further improvements are needed. First, the sample size of this retrospective study was relatively small, despite being the maximum in TED radiomics research works published to date. Thus, a larger sample size is expected to augment the reliability. Second, our models lack external validation. As TED management is highly complicated, the judgement of the activity of patients varies widely among centers, with different parameters for clinical measurements and MRI data acquisition, which makes it extremely challenging to integrate. This could potentially be tackled in the future by conducting a multicenter prospective study with unified metrics. While our study provides a new strategy for future research in this area, it is important to consider these limitations when interpreting our results.

## Conclusions

The results of this study revealed that radiomics models based on the whole orbital structures can accurately predict the response to IVGC in TED patients with the highest AUC of 0·961. Therefore, the MRR model is a reliable and effective tool for outcome prediction. The FRR model performed very well in reducing the time consumption of segmentation while preserving a rather satisfactory prediction value; thus, it can be applied as an alternative. The findings of our study could considerably contribute to the accurate prediction of responsive or unresponsive TED patients and allow for individualized management and therapy decisions, leading to improved patient prognosis and quality of life. In the meantime, the WOR strategy can be generalized to the application of other orbital diseases.

### Supplementary Information

**Additional file 1: Fig. S1.** The flowchart of patient enrollment and scheme for analysis. **Fig. S2. **Performances of SIR models using six machine learning algorithms in the test cohort were evaluated and compared through ROC curves. **Table S1.** The Rad score formula used in model performance. **Table S2.** Diagnostic performance of different SRR models.

## Data Availability

The datasets generated and analyzed during the current study are available by the corresponding author Huifang Zhou upon reasonable request.

## Abbreviations

AUC: Area under curve
BCVA: Best corrected visual acuity
CAS: Clinical activity score
DCA: Decision curve analysis
DON: Dysthyroid optic neuropathy
EOMs: Extraocular muscles
ExtraTrees: Extremely randomized trees
FOS: Fused-organ segmentation
FRR: Fused-regional radiomics
GLCM: Gray-level co-occurrence matrix
GLRLM: Gray-level run length matrix
GLSZM: Gray-level size zone matrix
GO: Graves’ orbitopathy
ICC: Intraclass correlation coefficients
IOP: Intraocular pressure
IR: Inferior rectus
IVGC: Intravenous glucocorticoid
LASSO: Least absolute shrinkage and selection operator
LG: Lacrimal gland
LightGBM: Light gradient boosting machine
LR: Lateral rectus
LR: Logistic regression
ML: Machine learning
MOS: Multi-organ segmentation
MR: Medial rectus
MRI: Magnetic resonance imaging
mRMR: Max-relevance and min-redundancy
MRR: Multi-regional radiomics
NGTDM: Neighborhood gray-tone difference matrix
NPV: Negative predictive value
OF: Orbital fat
ON: Optic nerve
PPV: Positive predictive value
ROC: Receiver operating characteristic
ROIs: Regions of interest
SI: Signal intensity
SIR: Signal intensity ratio
SO: Superior oblique
SR: Superior rectus
SRR: Single-regional radiomics
SVM: Support vector machines
T2-DRIVE: Coronal T2-weighted Turbo Spin-Echo with 90° Flip-Back Pulse
T2WI: T2-weighted imaging
TAO: Thyroid-associated ophthalmopathy
TED: Thyroid eye disease
TRAb: Thyroid-stimulating hormone receptor antibodies
WOR: Whole-orbit radiomics
XGBoost: Extreme gradient boosting
