# Supplementary material for: Whole-orbit radiomics: machine learning-based multi- and fused- region radiomics signatures for intravenous glucocorticoid response prediction in thyroid eye disease
Source: J Transl Med. 2024 Jan 13;22:56. doi: 10.1186/s12967-023-04792-2 (PMC10787992; doi:10.1186/s12967-023-04792-2)
Supplement: Supplementary file 1 — Additional file 1: Fig. S1. The flowchart of patient enrollment and scheme for analysis. Fig. S2. Performances of SIR models using six machine learning algorithms in the test cohort were evaluated and compared through ROC curves. Table S1. The Rad score formula used in model performance. Table S2. Diagnostic performance of different SRR models. [file 12967_2023_4792_MOESM1_ESM.pdf]

Whole-Orbit Radiomics: Machine Learning-Based Multi- and Fused-Region Radiomics Signatures for Intravenous Glucocorticoid Response Prediction in Thyroid Eye Disease

Haiyang Zhang[1,2, †], Mengda Jiang[3, †], Hoi Chi Chan[1,2], Huijie Zhang[1,2], Jiashuo Xu[1,2], Yuting Liu[1,2], Ling Zhu[3], Xiaofeng Tao[3], Duojin Xia[4], Lei Zhou[4], Yinwei Li[1,2], Jing Sun[1,2], Xuefei Song[1,2*], Huifang Zhou[1,2*], Xianqun Fan[1,2*]

[†]Haiyang Zhang and Mengda Jiang have contributed equally to this work and share first authorship.

*Correspondence: Xianqun Fan, fanxq@sjtu.edu.cn; Huifang Zhou, fangzzfang@sjtu.edu.cn; Xuefei Song, songxuefei@shsmu.edu.cn

[1]Department of Ophthalmology, Shanghai Ninth People's Hospital, Shanghai Jiao Tong University School of Medicine, Shanghai, China

[2]Shanghai Key Laboratory of Orbital Diseases and Ocular Oncology, Shanghai, China

[3]Department of Radiology, Shanghai Ninth People's Hospital, Shanghai Jiao Tong University School of Medicine, Shanghai, China

[4]School of Health Science and Engineering, University of Shanghai for Science and Technology, Shanghai, China

SUPPLEMENTARY MATERIAL---3

Logistics Regression--3

NaiveBayes---3

Support Vector Machine --3

ExtraTrees, Xgboost and LightGBM ---3

SUPPLEMENTARY FIGURES---5

Fig. 1 The flowchart of patient enrollment and scheme for analysis.--5

Fig. 2 Performances of SIR models using six machine learning algorithms in the test cohort were evaluated and compared through ROC curves.--6

SUPPLEMENTARY TABLE --7

Table 1. The Rad score formula used in model performance. --7

Table 2. Diagnostic performance of different SRR models. ---8

REFERENCE: --9

Supplementary Material

Logistics Regression

Logistic regression is a linear regression model that assumes the data follows a Bernoulli distribution. It utilizes the maximum likelihood function and gradient descent method to estimate the parameters, achieving binary classification. The logistic regression model can be seen as a linear regression model that is normalized by the Sigmoid function (also known as the Logistic equation) [1]. The Sigmoid function compresses the output of the linear regression (referred to as the logit) between [0, 1] and passes through the important point (0, 0.5). This compression ensures that the output is bounded between [0, 1], with a threshold of 0.5. Values greater than 0.5 are classified as one class, while values less than 0.5 are classified as the other class [2]. During the model development, the tuning step involves the use of "C", "penalty", and "solver" parameters.

NaiveBayes

NaiveBayes is a popular and simple probabilistic machine learning algorithm used for classification tasks. It is based on Bayes' theorem and assumes independence among the features of the input data, hence the term "naive." [3] The algorithm calculates the probability of a sample belonging to each class and assigns it to the class with the highest probability [4]. Naive Bayes utilizes prior knowledge of the class distribution and the likelihood of observing certain features to make predictions.

Support Vector Machine

Support Vector Machine (SVM) is a powerful and widely used supervised machine learning algorithm for both classification and regression tasks [5]. SVM is particularly effective in dealing with complex and high-dimensional datasets. The fundamental principle of SVM is to find an optimal hyperplane that separates different classes in the feature space. It aims to maximize the margin between the classes, which is the distance between the hyperplane and the nearest data points from each class. These data points, known as support vectors, play a crucial role in defining the decision boundary [6].

ExtraTrees, Xgboost and LightGBM

Random forests are ensemble models consisting of multiple decision trees [7]. They can be used for both classification and regression tasks, including multi-class classification. Random forests introduce nonlinearity and randomness into the model. In classification, each decision tree predicts the test sample, and the final prediction is determined by majority voting. Unlike traditional decision trees, random forests randomly select features from a subset during node splitting, instead of considering all features. This randomness slightly increases the bias but reduces the variance by averaging the predictions, resulting in an improved overall model.

The Extra-Trees (Extremely randomized trees) method is similar to random forests and often considered a variant of them [8]. However, Extra-Trees exhibit even greater randomness in the splitting of decision tree nodes. Each decision tree in Extra-Trees directly uses a random feature and threshold for splits, leading to more diversity between submodels. This increased randomness helps suppress overfitting, as extreme data points have less influence due to the high variability among decision trees. By preventing overfitting, Extra-Trees reduce variance while potentially increasing bias.

LightGBM was introduced as an optimization over XGBoost, a popular gradient boosting decision tree (GBDT) tool [9]. LightGBM aims to accelerate GBDT model training without compromising accuracy through several optimizations. It utilizes a histogram-based decision tree algorithm and implements Gradient-based One-Side Sampling (GOSS) to reduce the number of instances with small gradients, saving time and space during information gain calculation. Exclusive Feature Bundling (EFB) is employed to bundle mutually exclusive features, reducing dimensionality [10]. LightGBM adopts a

leaf-wise leaf growth strategy with depth limit, which improves efficiency compared to the traditional level-wise strategy. It directly supports categorical features, enables efficient parallelism, and optimizes cache hit rate. These optimizations make LightGBM a powerful and efficient GBDT tool.

Supplementary Figures

Fig. 1 The flowchart of patient enrollment and scheme for analysis.

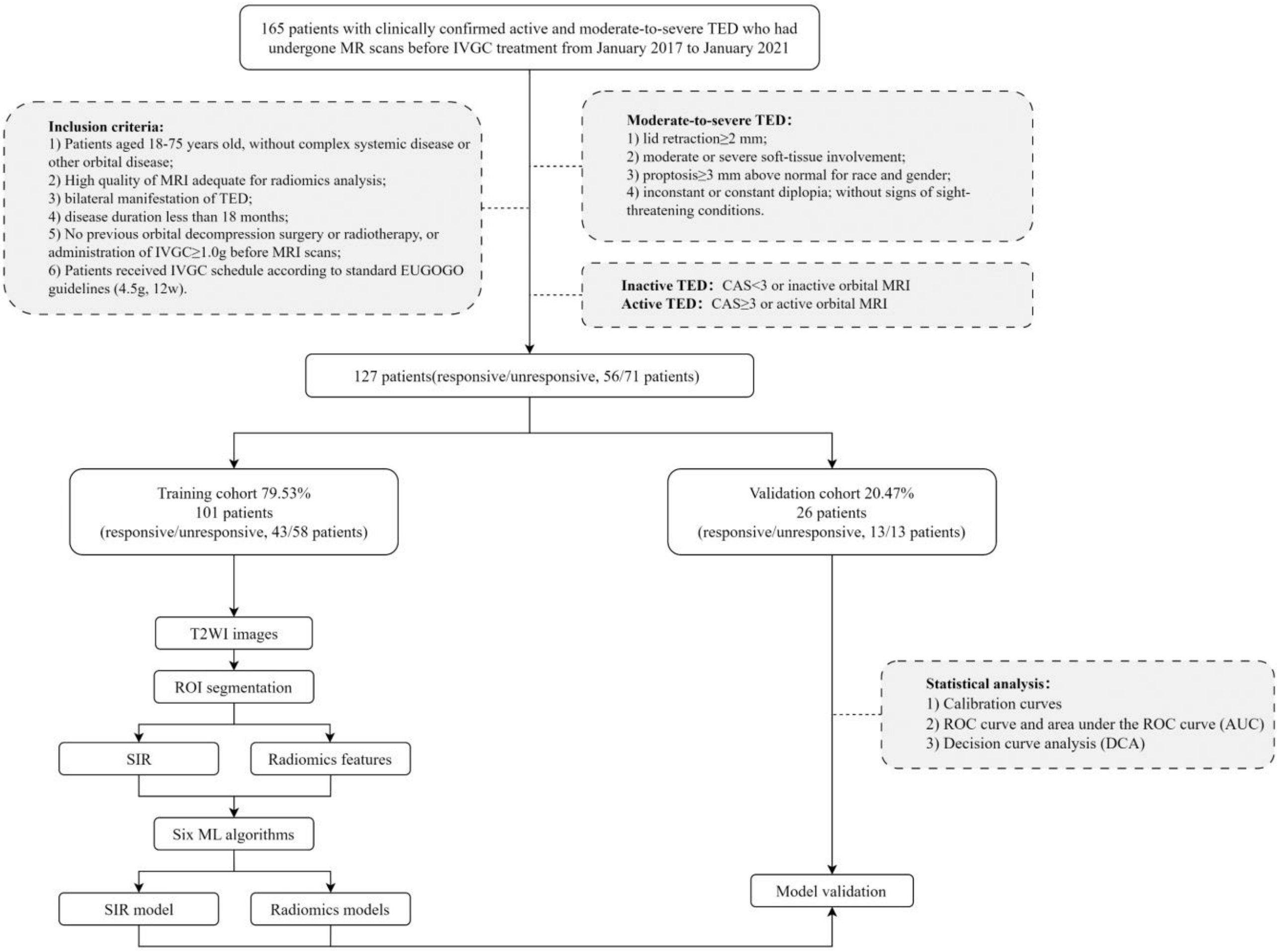

(TED, thyroid eye disease; T2WI, T2-weight imaging; ROI, region of interest; SIR, signal intensity ratio; ML, machine learning; ROC, receiver operator characteristic curve; AUC, area under curve)

Fig. 2 Performances of SIR models using six machine learning algorithms in the test cohort were evaluated and compared through ROC curves.

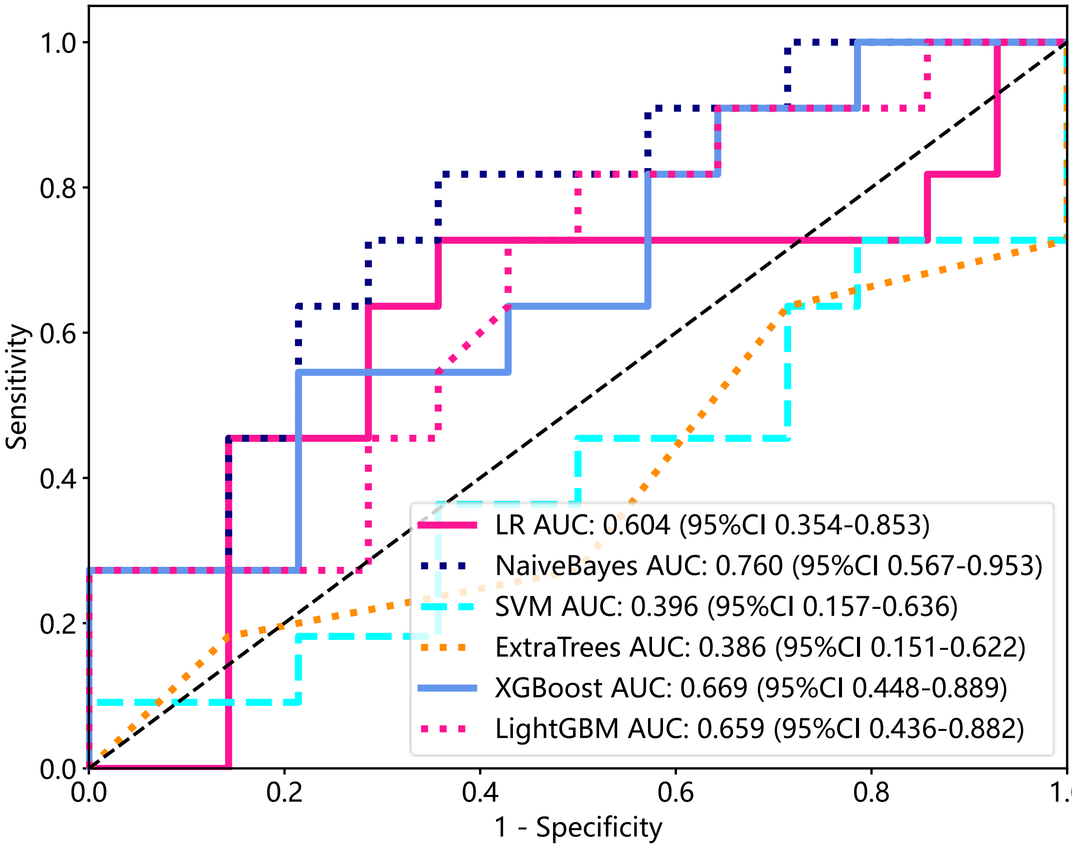

(SIR, signal intensity ratio; ROC, receiver operator characteristic curve; AUC, area under curve; LR, logistic regression; SVM, support vector machines; ExtraTrees, extremely randomized trees; XGBoost, extreme gradient boosting; LightGBM, light gradient boosting machine)

Supplementary Table

Table 1. The Rad score formula used in model performance.

	Rad score formula
**EOMs Radiomics model**	label = 0.45 + 0.069223 * wavelet_HHL_glszm_ZoneVariance_EOM + 0.090701 * wavelet_LHH_glcm_ClusterShade_EOM + 0.003204 * log_sigma_5_0_mm_3D_firstorder_Skewness_EOM + 0.009490 * original_shape_Maximum2DDiameterRow_EOM + 0.076605 * wavelet_HHH_glcm_Idm_EOM + 0.024620 * log_sigma_5_0_mm_3D_glcm_ClusterShade_EOM - 0.098575 * wavelet_LLL_firstorder_Minimum_EOM
**LG Radiomics model**	label = 0.44999999999999574 + 0.034796 * wavelet_LHH_firstorder_Median_LG + 0.076734 * wavelet_HLH_firstorder_Mean_LG -0.039490 * lbp_3D_m2_glcm_ClusterShade_LG - 0.115765 * wavelet_LLH_firstorder_Maximum_LG + 0.085380 * wavelet_HHL_glcm_Idm_LG + 0.136803 * exponential_glszm_SmallAreaEmphasis_LG + 0.058475 * wavelet_HLH_glszm_GrayLevelVariance_LG - 0.105635 * gradient_glrlm_ShortRunEmphasis_LG
**ON Radiomics model**	label = 0.45000000000000057 + 0.060017 * log_sigma_3_0_mm_3D_glszm_GrayLevelNonUniformity_ON + 0.084836 * wavelet_LHL_glcm_Idn_ON - 0.046791 * lbp_3D_m2_glrlm_RunPercentage_ON + 0.094455 * wavelet_LLL_glcm_DifferenceVariance_ON - 0.065940 * squareroot_glrlm_ShortRunLowGrayLevelEmphasis_ON - 0.060861 * exponential_gldm_DependenceNonUniformityNormalized_ON
**OF Radiomics model**	label = 0.4500000000000001 - 0.051348 * wavelet_HHL_firstorder_Mean_OF + 0.073616 * log_sigma_5_0_mm_3D_firstorder_Skewness_OF + 0.070924 * square_glszm_LargeAreaEmphasis_OF - 0.006614 * wavelet_LLL_glcm_ClusterShade_OF + 0.057661 * lbp_3D_m1_glszm_SizeZoneNonUniformity_OF
**MRR model**	label = 0.44999999999998325 + 0.005600 * log_sigma_3_0_mm_3D_glszm_GrayLevelNonUniformity_ON + 0.012353 * wavelet_LHL_firstorder_Skewness_MR + 0.046109 * wavelet_LLH_glszm_SmallAreaHighGrayLevelEmphasis_MR + 0.019191 * log_sigma_5_0_mm_3D_firstorder_TotalEnergy_IR + 0.107160 * wavelet_LLL_glcm_DifferenceVariance_ON + 0.023031 * wavelet_LHL_glcm_Idn_ON - 0.005761 * logarithm_firstorder_Skewness_LR + 0.034263 * log_sigma_5_0_mm_3D_firstorder_Skewness_OF - 0.020894 * lbp_3D_m2_glrlm_RunPercentage_ON + 0.046937 * wavelet_LHL_glcm_Idn_IR + 0.099162 * wavelet_HHL_firstorder_90Percentile_LR + 0.059301 * log_sigma_2_0_mm_3D_glszm_ZoneVariance_LR + 0.023941 * log_sigma_2_0_mm_3D_firstorder_RootMeanSquared_IR - 0.055326 * gradient_glszm_ZoneEntropy_ON - 0.028953 * square_glszm_ZoneVariance_MR + 0.002952 * square_glcm_InverseVariance_MR - 0.047420 * exponential_glszm_SmallAreaLowGrayLevelEmphasis_MR + 0.027035 * exponential_glszm_ZoneVariance_ON + 0.055457 * wavelet_LHH_firstorder_Median_LG + 0.046650 * lbp_3D_k_glcm_Imc2_LR + 0.027695 * wavelet_HLL_glszm_SmallAreaHighGrayLevelEmphasis_LR + 0.084323 * square_glszm_LargeAreaEmphasis_OF + 0.050349 * exponential_glcm_Autocorrelation_ON - 0.021314 * lbp_3D_m2_glrlm_RunLengthNonUniformityNormalized_SO - 0.052108 * lbp_3D_k_glcm_Correlation_IR + 0.032520 * log_sigma_3_0_mm_3D_glrlm_GrayLevelNonUniformity_IR + 0.041683 * wavelet_HLH_glcm_Idn_ON + 0.064903 * wavelet_HLL_glrlm_ShortRunEmphasis_MR + 0.020022 * gradient_gldm_LargeDependenceHighGrayLevelEmphasis_ON +0.024759 * wavelet_HHH_glrlm_GrayLevelNonUniformity_ON -0.004454 * squareroot_glcm_ClusterShade_IR + 0.024691 * original_shape_MajorAxisLength_SR + 0.025217 * wavelet_HHL_glszm_GrayLevelNonUniformity_SO + 0.035932 * log_sigma_3_0_mm_3D_glszm_ZoneVariance_LR - 0.056570 * wavelet_LLH_firstorder_Maximum_LG
**FRR model**	label = 0.4500000000000006 - 0.127778 * log_sigma_4_0_mm_3D_glcm_MaximumProbability_Whole - 0.071556 * squareroot_glszm_LargeAreaLowGrayLevelEmphasis_Whole -0.087967 * wavelet_LLL_firstorder_Minimum_Whole - 0.085919 * square_glszm_SmallAreaEmphasis_Whole + 0.058320 * lbp_3D_k_firstorder_Minimum_Whole + 0.047042 * log_sigma_5_0_mm_3D_glcm_ClusterShade_Whole + 0.096652 * wavelet_HHH_firstorder_Median_Whole + 0.059793 * log_sigma_2_0_mm_3D_glcm_MaximumProbability_Whole

Table 2. Diagnostic performance of different SRR models.

SRR models	ML	AUC	Accuracy	Sensitivity	Specificity	PPV	NPV
EOM radiomics model	LR	0.701	0.600	0.545	0.643	0.545	0.643
	NaiveBayes	0.708	0.680	0.636	0.714	0.636	0.714
	SVM	0.695	0.680	0.545	0.786	0.667	0.688
	ExtraTrees	0.679	0.640	0.545	0.714	0.600	0.667
	XGBoost	0.766	0.680	0.545	0.786	0.667	0.688
	LightGBM	0.685	0.600	0.364	0.786	0.571	0.611
LG radiomics model	LR	0.643	0.520	0.455	0.571	0.455	0.571
	NaiveBayes	0.675	0.640	0.545	0.714	0.600	0.667
	SVM	0.656	0.600	0.545	0.643	0.545	0.643
	ExtraTrees	0.529	0.520	0.455	0.571	0.455	0.571
	XGBoost	0.552	0.520	0.455	0.571	0.455	0.571
	LightGBM	0.607	0.560	0.545	0.571	0.500	0.615
OF radiomics model	LR	0.766	0.760	0.727	0.786	0.727	0.786
	NaiveBayes	0.760	0.680	0.727	0.643	0.615	0.750
	SVM	0.701	0.640	0.182	1.000	1.000	0.609
	ExtraTrees	0.698	0.680	0.455	0.857	0.714	0.667
	XGBoost	0.740	0.640	0.545	0.714	0.600	0.667
	LightGBM	0.714	0.640	0.636	0.643	0.583	0.692
ON radiomics model	LR	0.649	0.560	0.455	0.643	0.500	0.600
	NaiveBayes	0.669	0.600	0.636	0.571	0.538	0.667
	SVM	0.610	0.680	0.545	0.786	0.667	0.688
	ExtraTrees	0.562	0.560	0.455	0.643	0.500	0.600
	XGBoost	0.662	0.600	0.455	0.714	0.556	0.625
	LightGBM	0.643	0.680	0.545	0.786	0.667	0.688

Reference:

1. Tolles J, Meurer WJ. Logistic Regression: Relating Patient Characteristics to Outcomes. JAMA. 2016;316(5):533–4.

2. Ng A. CS229 Lecture notes. CS229 Lecture notes. 2000;1(1):1–3.

3. Russell SJ. Artificial intelligence a modern approach. Pearson Education, Inc.; 2010.

4. Murty MN, Devi VS. Pattern recognition: An algorithmic approach. Springer Science & Business Media; 2011.

5. Cortes C, Vapnik V. Support-vector networks. Machine learning. 1995;20:273–97.

6. Joachims T. Text categorization with support vector machines: Learning with many relevant features. In: Machine Learning: ECML-98: 10th European Conference on Machine Learning Chemnitz, Germany, April 21–23, 1998 Proceedings. Springer; 2005. p. 137–42.

7. Ho TK. Random decision forests. In: Proceedings of 3rd international conference on document analysis and recognition. IEEE; 1995. p. 278–82.

8. Geurts P, Ernst D, Wehenkel L. Extremely randomized trees. Machine learning. 2006;63:3–42.

9. Mehta M, Agrawal R, Rissanen J. SLIQ: A fast scalable classifier for data mining. In: Advances in Database Technology—EDBT'96: 5th International Conference on Extending Database Technology Avignon, France, March 25–29, 1996 Proceedings 5. Springer; 1996. p. 18–32.

10. Ke G, Meng Q, Finley T, Wang T, Chen W, Ma W, et al. LightGBM: a highly efficient gradient boosting decision tree. In: Proceedings of the 31st International Conference on Neural Information Processing Systems. Red Hook, NY, USA: Curran Associates Inc.; 2017. p. 3149–57. (NIPS'17).
